# Delayed luminescence guided enhanced circularly polarized emission in atomically precise copper nanoclusters†

**DOI:** 10.1039/d3sc00686g

**Published:** 2023-04-25

**Authors:** Camelia Dutta, Sonia Maniappan, Jatish Kumar

**Affiliations:** a Department of Chemistry, Indian Institute of Science Education and Research (IISER) Tirupati Tirupati – 517507 India jatish@iisertirupati.ac.in

## Abstract

Metal nanoclusters, owing to their intriguing optical properties, have captivated research interest over the years. Of special interest have been chiral nanoclusters that display optical activity in the visible region of the electromagnetic spectrum. While the ground state chiral properties of metal nanoclusters have been reasonably well studied, of late research focus has shifted attention to their excited state chiral investigations. Herein, we report the synthesis and chiral investigations of a pair of enantiomerically pure copper nanoclusters that exhibit intense optical activity, both in their ground and excited states. The synthesis of nanoclusters using l- and d-isomers of the chiral ligand led to the formation of metal clusters that displayed mirror image circular dichroism and circularly polarized luminescence signals. Structural validation using single crystal XRD, powder XRD and XPS in conjunction with chiroptical and computational analysis helped to develop a structure–property correlation that is unique to such clusters. Investigations on the mechanism of photoluminescence revealed that the system exhibits long excited state lifetimes. A combination of delayed luminescence and chirality resulted in circularly polarized delayed luminescence, a phenomenon that is rather uncommon to the field of metal clusters. The chiral emissive properties could be successfully demonstrated in free-standing polymeric films highlighting their potential for use in the field of data encryption, security tags and polarized light emitting devices. Moreover, the fundamental understanding of the mechanism of excited state chirality in copper clusters opens avenues for the exploration of similar effects in a variety of other clusters.

## Abstract 2

Chiral copper clusters displaying good luminescence and extended lifetimes were synthesised. Enantiomerically pure clusters capped using l- and d-isomers of the ligand displayed mirror image circular dichroism and circularly polarized luminescence.

## Graphical abstract

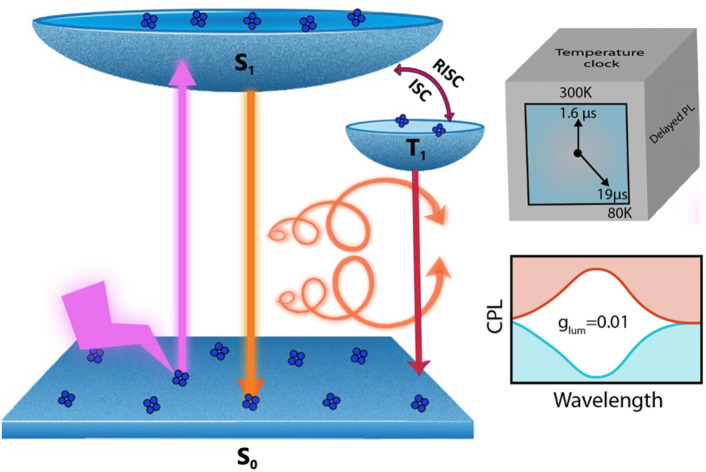

## Introduction

Metal nanoclusters that are typically of size less than 2 nm act as the missing link between molecules and nanoparticles.^1–3^ Since the observation of intriguing properties in these atomically precise clusters, research focused on these materials has attracted considerable interest from scientists across the globe.^4–6^ The exciting properties exhibited by these nanomaterials can be attributed to the presence of quantized and discrete energy levels, and the well-defined electronic transitions between them. Major research focus has been on understanding the role of atomic level structural parameters on the observed photophysical properties thereby drawing a concrete structure–property correlation.^7^ Among the different metal nanoclusters investigated, copper clusters are of special interest, not only due to the abundance of raw materials, ease of synthesis and appealing physico-chemical properties, but also due to their potential application in the fields of catalysis, electronics and biosensing.^8–13^ Unlike nanoclusters based on noble metals such as platinum,^14^ gold,^15,16^ and silver,^17^ the synthesis and isolation of copper nanoclusters remain a challenge due to the unstable nature of copper ions towards air oxidation. However, the use of surface capping ligands possessing desired functionalities can bind to the metal and act as the protecting layer resulting in robust clusters.^18–21^ Moreover, owing to their rigid structural backbone, copper nanoclusters display superior properties such as thermal and photochemical stability. As a result, technological applications of these materials in emerging fields such as optical devices, surface coatings and light-emitting devices are being widely explored.^22,23^

**Fig. 1:**
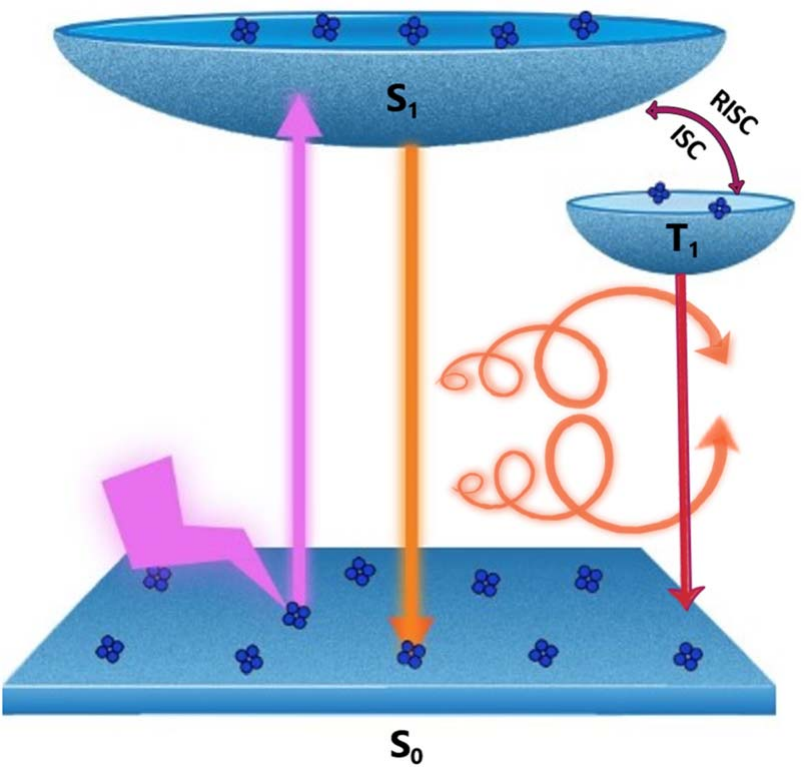
Scheme illustrating the various electronic transitions during CP-TADF and phosphorescence in copper nanoclusters.

Chiral clusters constitute an important subgroup in metal nanoclusters as they display distinct optical responses compared to their achiral counterparts. While ground state chirality in nanoclusters is reasonably well explored,^24–27^ investigations on their excited state optical activity is still in its infancy. A combination of optical activity and luminescence can lead to an exciting property known as circularly polarized luminescence (CPL), which refers to the differential emission of left- and right-circularly polarized light.^28–34^ In addition to providing valuable inputs on the excited state chiral properties in materials, owing to its widespread potential in the fields of chiral light emitting materials, information storage, and encrypted transmission, CPL has emerged as a promising area of research in the last decade.^35–38^ Even though CPL has been extensively used to study the excited state chiral properties of organic and inorganic molecular systems,^39–41^ the technique is relatively new to the field of luminescent nanomaterials.^42–44^ Among the various mechanisms of CPL reported, circularly polarised thermally activated delayed fluorescence (CP-TADF) has emerged as a promising candidate due to its ability to harvest triplet excitons and thereby enhance the efficiency in light emitting diodes.^45–47^ CP-TADF is reported in a few organic systems possessing a close singlet–triplet energy gap;^48,49^ however, such an observation is relatively new to the field of nanocluster chirality. We herein report a pair of cysteine capped copper nanoclusters [(d/l-Cys)_2_Cu_4_I_4_] that exhibit long excited state lifetimes. The clusters, due to a close singlet–triplet energy gap, undergo reverse intersystem crossing (RISC) resulting in a relatively high photoluminescence quantum yield (PLQY) and intense CPL activity (Fig. 1). These properties could be successfully demonstrated both in the solution state and solid films.

## Results and discussion

### Structural characterization of nanoclusters

**Fig. 2:**
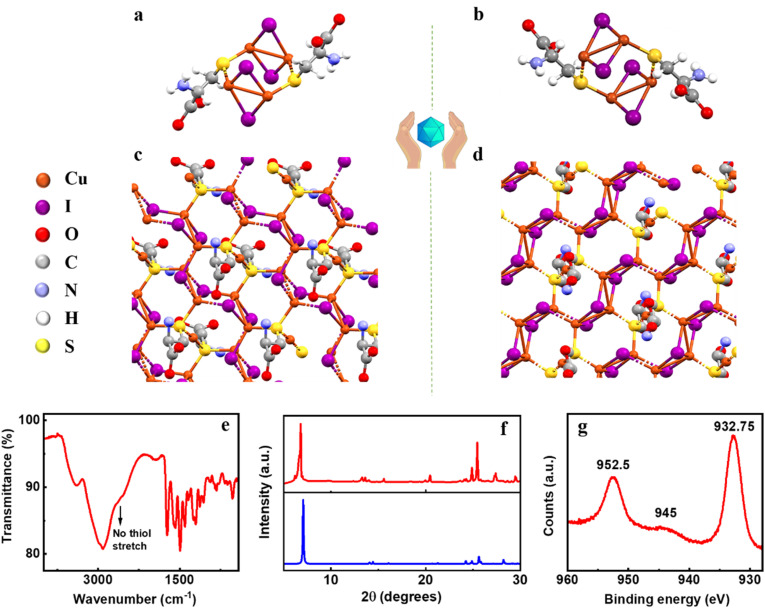
X-ray crystal structure of (a) (d-Cys)_2_Cu_4_I_4_ and (b) (l-Cys)_2_Cu_4_I_4_ nanoclusters and (c and d) their corresponding extended network structures. (e) FTIR spectrum, (f) simulated (blue trace) and experimental (red trace) powder XRD plots, and (g) XPS spectrum of the (d-Cys)_2_Cu_4_I_4_ nanocluster sample.

The two isomers of the nanoclusters were synthesized by reacting CuI solution with d/l-cysteine at room temperature. The system was incubated overnight in the dark to form bright yellow-coloured nanoclusters. Centrifugation and repeated washing were carried out to remove excess reagents. Single crystal X-ray diffraction (SC-XRD) is a powerful technique for the structural characterization of nanoclusters. Dissolving the purified samples of d- and l-isomer in chloroform and acetone, respectively, followed by slow evaporation yielded bright yellow single crystals of the enantiomeric clusters. XRD data collected for both the clusters revealed that (d-Cys)_2_Cu_4_I_4_ and (l-Cys)_2_Cu_4_I_4_ crystallize in C2 (monoclinic) and P1 (triclinic) space groups, respectively (Fig. 2a and b). The two binuclear Cu–I cores in the center are attached to two sulphur atoms of the cysteine units. For each binuclear Cu–I core, the Cu is attached to the cysteine ligand through a sulphur atom which is further attached to the other binuclear Cu–I core through the Cu atom. The binuclear Cu–I core adopts a planar geometry with μ^2^ iodides, whereas the sulphur atom along with the cysteine residue is positioned in a different plane. μ^2^ iodides are coordinated with two adjacent Cu atoms with a 2.6 Å Cu–I bond distance. The monomeric crystal structure of (d-Cys)_2_Cu_4_I_4_ and (l-Cys)_2_Cu_4_I_4_ reveals the presence of two cysteine residues as the contributing groups to the origin of chirality in the nanoclusters (*vide infra*). The crystal further showed the propensity of growth in different directions resulting in the formation of coordinated network structures (Fig. 2c and d). Further details on the crystallographic parameters are provided in the ESI (Table S1†).

To establish the sample purity and to thoroughly characterize the clusters, additional characterization techniques like Fourier-transform infrared spectroscopy (FTIR), X-ray photoelectron spectroscopy (XPS) and powder X-ray diffraction (PXRD) were employed. The FTIR spectrum of the nanoclusters exhibited a broad band at 3398 cm^−1^ corresponding to the symmetric and asymmetric amine stretching (Fig. 2e). The broad band near 3000 cm^−1^ can be ascribed to the C–H and O–H stretching of the carboxylic acid group. The absence of a sharp peak in the 2600–2550 cm^−1^ range is indicative of the disappearance of the free thiol group. This corroborates well with the SC-XRD data (*vide supra*) and supports the hypothesis that the sulphur atom is attached to the metal center. Sharp peaks at 1728 cm^−1^ and 1406 cm^−1^ represent the C=O stretching and O–H bending of the carboxylic functional group, respectively. The peaks present in the range of 1250–1032 cm^−1^ are ascribed to the C–N stretching in the clusters. Zeta potential measurements showed a positive surface charge of +34.1 mV and +30.7 mV for the d- and l-isomers of the nanoclusters, respectively (Fig. S1†). The excess positive charge over the cluster surface could be attributed to the existence of the ionized state (NH_3_^+^) of the amino acids at acidic pH. The experimental PXRD data corroborate well with the PXRD pattern simulated from the single crystal data confirming the purity of the analysed samples (Fig. 2f). To investigate the oxidation state of Cu, XPS analysis of the samples was carried out. XPS spectra show peaks at 932.75 eV and 952.5 eV corresponding to Cu 2p_3/2_ and Cu 2p_1/2_, respectively (Fig. 2g). A weak satellite peak around 945 eV suggests the oxidation state of copper as Cu(i).

### Optical properties of the chiral nanoclusters

**Fig. 3:**
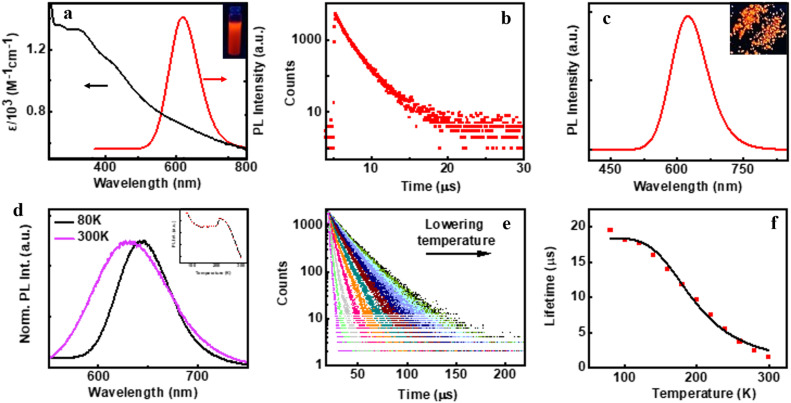
(a) UV-visible absorption (black trace), PL emission (red trace) and (b) PL lifetime decay plot of aqueous (d-Cys)_2_Cu_4_I_4_ nanocluster solution. (c) Emission spectra of the (d-Cys)_2_Cu_4_I_4_ single crystal sample. Inset in (a) and (c) shows the photographic images of nanocluster solution and single crystals, respectively, under UV light illumination. Temperature dependent (d) normalized luminescence spectra and (e) PL lifetime decay plot of the crystals. Inset in (d) shows the temperature dependent changes in PL intensity. (f) PL lifetime decay *versus* temperature plot fitted with the Boltzmann equation.

The preliminary photophysical investigations of the clusters were carried out using UV-visible and fluorescence spectroscopy (Fig. 3a). Both isomers of the Cu clusters exhibited broad absorption spectra with peaks centred around 310 and 431 nm as reported on similar clusters.^50^ The photoluminescence (PL) spectra of enantiomeric nanoclusters dispersed in solution exhibited a strong orange emission at room temperature. The spectra showed a maximum emission intensity centred at 625 nm with a PLQY of 10.83% and 10.50% for (d-Cys)_2_Cu_4_I_4_ and (l-Cys)_2_Cu_4_I_4_ clusters, respectively. The PL lifetime calculated using multi-channel scaling (MCS) experiments on both the isomers in solution showed fits with a biexponential decay function (Fig. 3b). The average lifetime was found to be 1.58 μs and 1.50 μs, for the enantiomeric d- and l-nanoclusters, respectively (Fig. 3b and S2a†). The crystals formed from the nanoclusters exhibited intense luminescence under UV illumination (Fig. 3c inset). Analogous to the spectral features in solution, the crystals formed from the nanoclusters exhibited intense red emission at 628 nm with a PLQY of 14.03% (Fig. 3c). The decay profile for the purified crystals showed a biexponential fitting with an average lifetime of 1.61 μs at room temperature. Such high lifetimes are indicative of spin forbidden transitions and when coupled with high PLQY is of special interest for application in the field of light emitting materials.

To further understand the excited state mechanism, we investigated the effect of temperature on the luminescence properties of the clusters. The PL spectra were collected at different temperatures ranging from 80 K to 363 K. A strong luminescence quenching was observed at higher temperature which could be regained on cooling down the system. The lowering of temperature also resulted in a redshift in the emission maxima from 628 nm to 645 nm in the 300–80 K range (Fig. 3d). Such a drastic change at lower temperature inspired us to investigate the temperature dependent changes in the excited state lifetimes. The lifetime plots of the solid crystals were collected under a nitrogen environment from 80 K to room temperature (Fig. 3e). The crystals exhibited a biexponential decay fitting with an average lifetime of 1.52 μs at room temperature. The lifetime increased to 19.53 μs at 80 K suggesting the possibility of a thermally activated delayed fluorescence (TADF) phenomenon. TADF is a highly efficient photophysical process wherein at a higher temperature RISC occurs from the lowest triplet energy state (T_1_) to the first singlet excited state (S_1_).^51^ This in turn results in an emission from the S_1_ to the S_0_ state instead of T_1_ to S_0_ decay, thus resulting in an increased lifetime (Fig. 1).^52,53^ In contrast, such opposite intersystem crossing is prohibited at a low temperature due to the lack of thermal activation energy. Thus, low energy separation between S_1_ and T_1_ states is a prerequisite for TADF to occur. To establish the observed effect as originating from TADF, we calculated the singlet–triplet energy separation in Cu nanoclusters by following the Boltzmann equation derived from the TADF model given below:^54,55^
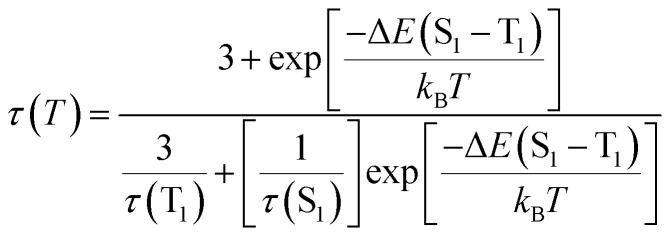
where *τ*(*T*), Δ*E*(S_1_ − T_1_), *τ*(T_1_), *τ*(S_1_), *k*_B_ and, *T* represent the lifetime (in μs) at temperature *T* (in K), energy separation between singlet (S_1_) and triplet states (T_1_), lifetime of the triplet state, lifetime of the singlet state, Boltzmann constant and absolute temperature, respectively. For effective TADF to occur, Δ*E*(S_1_ − T_1_) should preferably have a value less than 0.37 eV. The lifetime *vs.* temperature plot fitted with the above equation gave a singlet–triplet energy separation value of 0.09 eV with an *R*-square (COD) of 0.99 (Fig. 3f). The lifetime of singlet and triplet states was found to be 0.03 μs and 18.4 μs, respectively. The radiative rate constant of TADF (*k*(S_1_)) and phosphorescence (*k*(T_1_)) was calculated to be 3.33 × 10^6^ s^−1^ and 5.4 × 10^4^ s^−1^, respectively. The low singlet–triplet energy gap and all other calculated values point towards the occurrence of TADF in the nanocluster sample.^56–58^

**Fig. 4:**
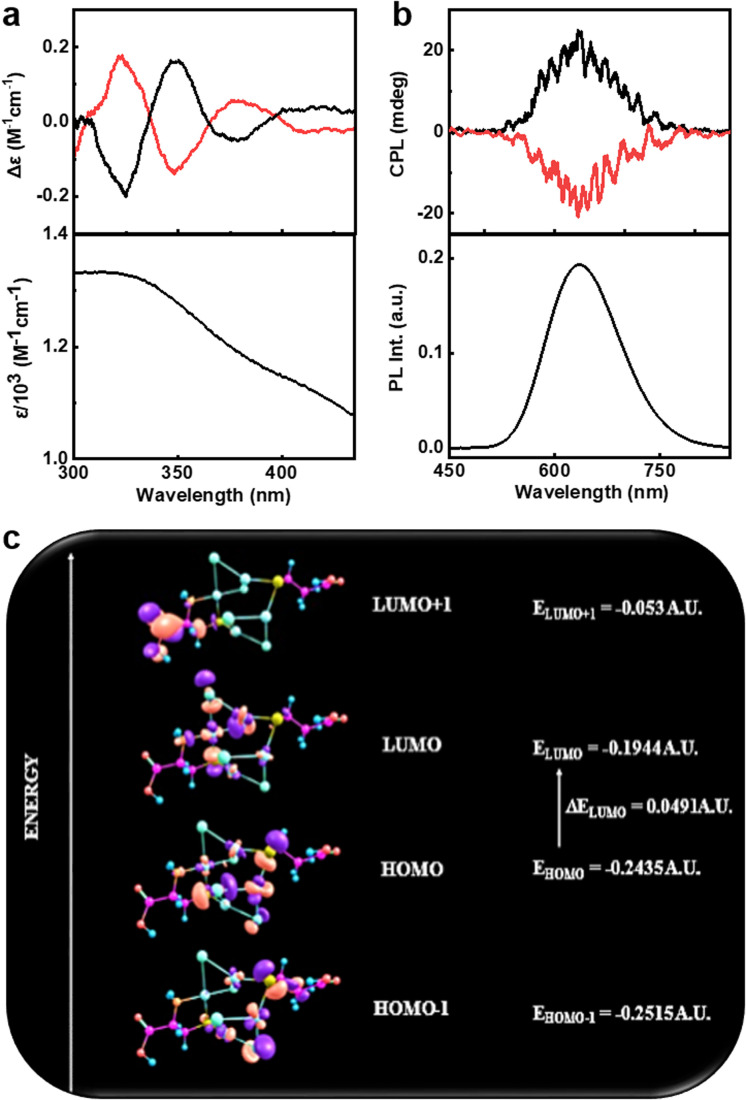
(a) Experimental CD spectra along with the corresponding absorption plot, and (b) experimental CPL spectra along with the corresponding luminescence spectra of the aqueous solution of nanoclusters. Black and red traces correspond to spectra collected from (d-Cys)_2_Cu_4_I_4_ and (l-Cys)_2_Cu_4_I_4_ nanoclusters, respectively. (c) Selected FMO representation for (Cys)_2_Cu_4_I_4_ in its optimised geometry.

The presence of cysteine molecules would provide chirality to the nanoclusters. In order to investigate the optical activity of the clusters, the circular dichroism (CD) and CPL spectra of the samples dispersed in water were analysed at room temperature. Ground state chiral investigations using CD spectroscopy showed mirror image profiles for (d-Cys)_2_Cu_4_I_4_ and (d-Cys)_2_Cu_4_I_4_ nanoclusters in the 300–450 nm spectral range (Fig. 4a and S3†). The CD profile matches well with the absorption peaks confirming the induction of optical activity into the corresponding transitions. To further understand the nature of these transitions, systematic theoretical simulations were performed on these clusters using Gaussian 09. The optimized structure obtained with the basis set def2tzvp and functional pbe1pbe is consistent with the crystal data (Fig. S4†). The simulated absorption spectra using time dependent density functional theory (TD-DFT) calculations considering 45 energy states showed intense absorption peaks at 310 and 433.5 nm corresponding to the transitions including excited states with energy 3.99 eV and 2.85 eV, respectively (Fig. S5a†). The simulated absorption spectra match well with the experimental plot indicating the structural consistency of the clusters in solution. The ECD spectrum obtained from theoretical calculations of (d-Cys)_2_Cu_4_I_4_ exhibited a similar pattern and sign to the experimental plot; however, there were deviations in the peak position, which could be attributed to the limited number of states adopted in simulations and the polydispersity of samples employed for experimental analysis (Fig. S5b†). The experimental and the theoretical data confirm the chiral induction in the prescribed transitions from the ground state. In addition to deriving the UV-vis and ECD spectra, TD-DFT was extended towards natural bond orbital (NBO) calculations with the same basis set and functional.^59^ NBO analysis can be used to obtain a clear description on the most possible ‘natural Lewis bond’ structure by calculating the highest probability of electron density. NBO studies revealed the nature of bonding within the system. Each Cu_2_I_2_ core consists of two 3 centered 2 electron (3c–2e) bonding (known as the banana bond), in which each bond possesses two Cu atoms and one I atom. In each 3c–2e bonding, the contribution of electronic density from iodine is highest. Moreover, iodine possesses a negative charge density whereas the charge over the Cu atoms is positive, confirming that iodine is acting as an electron donating center. Fig. 4c represents the frontier molecular orbital (FMO) diagram depicting the possible HOMO–LUMO levels for the observed transitions.

The strong PL from the nanoclusters facilitated the investigations on its excited state chiroptical properties using CPL spectroscopy. Interestingly, the enantiomeric nanoclusters exhibited intense CPL signals at the corresponding emission wavelength (Fig. 4b). Positive and negative CPL plots were obtained for the (d-Cys)_2_Cu_4_I_4_ and (l-Cys)_2_Cu_4_I_4_ nanoclusters, respectively. The extent of chiral dissymmetry in luminescence is calculated using the luminescence anisotropy factor (*g*_lum_) given by the equation *g*_lum_ = 2(*I*_L_ − *I*_R_)/(*I*_L_ + *I*_R_), where *I*_L_ and *I*_R_ are the intensities of left and right circularly polarized light, respectively.^60^ The d- and l-enantiomers of the nanoclusters exhibited high *g*_lum_ values of +1.22 × 10^−2^ and −1.00 × 10^−2^, respectively, at the emission maxima (Fig. S6a†). The luminescence anisotropy observed for the copper nanoclusters are almost an order magnitude higher when compared with the values reported on similar nanomaterials that exhibit chiral emission. Hence, the clusters exhibit enhanced chiral luminescence that is guided by the TADF effect. The sign of CPL signals matches well with the CD at the highest wavelength indicating that the chiral emission follows the same handedness as that of the absorption.

To further understand the evolution of chirality in the clusters, we monitored the progression of CPL and PL signals during the synthesis of the clusters. No CPL signals were observed from samples soon after the addition of the precursors (Fig. S7†). Weak CPL signals started appearing after 30 min which intensified further with time. The PL intensity increased gradually after 30 min and reached saturation at 3 h. We further investigated the stability of the signals at varying temperature and pH. The pH guided stability of the system was studied by monitoring the emission and CPL response of the nanoclusters at varying pH (Fig. S8†). The cluster solution was found to be most stable at an acidic pH of 1.5 (pH of the reaction medium), whereas increasing pH resulted in the broadening of absorption and CD signals. PL along with CPL signals quenched at higher pH values. While the PL could be fully regained on lowering the pH, the CPL could only be recovered partially, may be due to the structural distortion of the clusters during the pH change. Temperature dependent CPL plots showed trends in agreement with the PL profile (Fig. S9†). Analogous to PL quenching observed at high temperature, CPL signals also diminished upon heating the sample to elevated temperatures. However, the signals could be fully regained upon cooling the sample to room temperature offering the possibility of developing a thermal switch based on CPL.

**Fig. 5:**
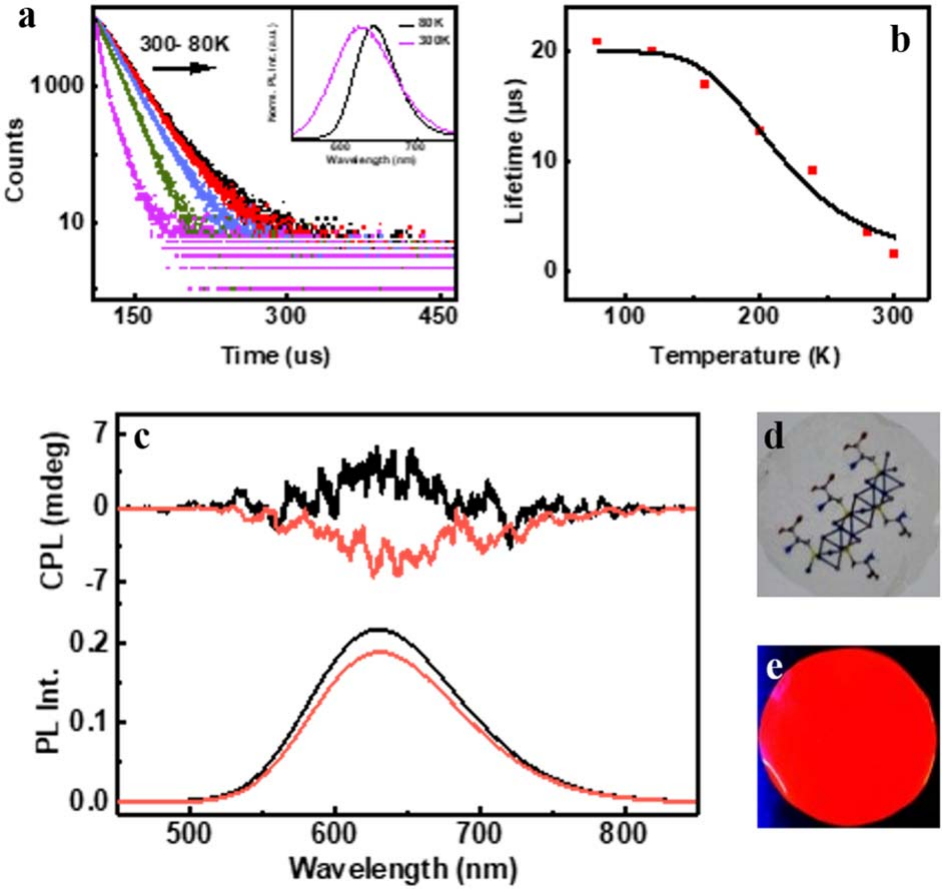
(a) Temperature dependent lifetime decay for the PVA film incorporated with (d-Cys)_2_Cu_4_I_4_. Inset shows the normalised PL spectra at 80 K and room temperature. (b) PL lifetime decay *vs.* temperature plot fitted with the Boltzmann equation. (c) CPL (top) and PL (bottom) spectra from the films prepared using (d-Cys)_2_Cu_4_I_4_ (black trace) and (l-Cys)_2_Cu_4_I_4_ (red trace) nanoclusters. Photographs of the film under (d) visible and (e) UV light illumination.

The intense PL and CPL activity along with the stability exhibited by the clusters have inspired us to fabricate chiral photonic films using the synthesized nanomaterials. Moreover, the demonstration of the observed chiroptical properties on the surface is rather important for any future application of the material. With an aim to display the properties on the surface, we have prepared a chiral transparent self-standing PVA film through an evaporation based self-assembly method. The nanocluster sample was added to an aqueous PVA solution in water under continuous stirring until mixed uniformly and converted into gel. The gel was drop-cast onto a Petri dish and kept in the dark at 35 °C for drying for 12 h to form transparent free-standing films that are orange emitting under a UV lamp (Fig. 5d and e). Due to the suppression of phonon-assisted nonradiative relaxation in the solid state, PLQY exhibited a considerable enhancement from 10.50% (in solution) to 16.32% in the film state. Through biexponential decay fitting, the average lifetime of the PVA film prepared using (d-Cys)_2_Cu_4_I_4_ was calculated to be 1.5 μs at room temperature (Fig. S10†). The high lifetime value prompted us to investigate the temperature dependent effects, studies similar to those carried out on the crystals. A redshift of 18 nm in PL emission was recorded on lowering the temperature (Fig. 5a inset). The lifetime increased to 20.78 μs upon decreasing the temperature to 80 K (Fig. 5a). The singlet–triplet energy separation was calculated from the lifetime *vs.* absolute temperature plot fitted with the Boltzmann equation (Fig. 5b). The energy separation Δ*E*(S_1_ − T_1_) was calculated to be 0.12 eV, which clearly shows that the observed luminescence at room temperature is a result of the TADF effect. The triplet and singlet lifetimes [*τ*(T_1_) and, *τ*(S_1_)] were found to be 19.92 and 0.0126 μs, respectively. The rate constant for the singlet [*k*(S_1_)] and triplet decay [*k*(T_1_)] was found to be 79.36 × 10^6^ s^−1^ and 5 × 10^4^ s^−1^, respectively. We further investigated the CPL properties of the films at room temperature. Interestingly, clear mirror image CPL signals with positive and negative signs were observed for the films prepared using (d-Cys)_2_Cu_4_I_4_ and (l-Cys)_2_Cu_4_I_4_, respectively (Fig. 5c). The d- and l-nanocluster films exhibited *g*_lum_ values of +2 × 10^−3^ and −2.25 × 10^−3^, respectively (Fig. S6b†). The sign of CPL in the films was retained as in the solution state indicating no structural change in the clusters upon incorporating into the polymeric films. CPL spectra were collected from different points by rotating and flipping the films (Fig. S11†). No considerable change in the *g*_lum_ values or the sign of signals was observed ruling out the possibility of any artefacts due to linear polarisation effects. Hence, the TADF based chiral emissive properties of the nanoclusters could be successfully established both in the solution state and solid films highlighting the potential of these nanoclusters in applications related to chiral light emitting devices and security tags.

From the experimental data it is evident that multiple factors contribute to the emission and in turn CPL of clusters from the excited state. The experimental data along with the reported literature on metal complexes and clusters point towards the contribution of both singlet and triplet states towards luminescence.^61–63^ The low singlet–triplet energy gap can facilitate the equilibration of the excitons between the singlet and triplet states and can aid the TADF effect at room temperature wherein enough thermal energy is available for the RISC.^64^ However, a contribution of phosphorescence towards the luminescence cannot be ruled out under these conditions. Moreover, there exists a possibility of the mixing of singlet and triplet states leading to excited states with a long lifetime. However, based on the calculated decay values from the temperature dependent lifetime studies and the observed shift in luminescence with decreasing temperature, we tentatively assign the broad emission at room temperature predominantly to the TADF effect with certain contribution from phosphorescence. In contrast, at low temperature where RISC is restricted, and the decay is primarily due to phosphorescence. The blue shift in the emission profile upon increasing the temperature corroborates with such a mechanism.^45,63,64^

## Conclusions

In summary, we have successfully designed and synthesized a new set of chiral transition metal-halide nanoclusters that exhibit high luminescence and optical activity. The structural characteristics of the nanoclusters established with the help of SC-XRD reveals an inner CuI core that is surrounded by a chiral cysteine ligand inducing optical activity in the clusters. The chiral handedness of the clusters is largely dependent on the direction of cysteine arrangement around the CuI core. This in turn governs the sign of chiral signals, both in its ground and excited state transitions. A combination of CPL and TADF gives rise to CP-TADF, an effect that is rather rare to the nanocluster systems. The demonstration of CP-TADF with enhanced luminescence and chiral anisotropy is of interest and may open new avenues for similar investigations on a vast variety of metal nanoclusters. Successful demonstration of such intriguing properties, both in solution and free-standing films, offers vast potential for these materials. The development of clusters with enhanced quantum yield can lead to commercialization of these materials in CP-LEDs and efforts are in progress in this direction.

## Data availability

All experimental details are added to the ESI file.†

## Author contributions

J. K. conceived and coordinated the project. C. D. and S. M. carried out the experiments. C. D. & J. K. analysed and consolidated the data. C. D. and J. K. prepared the manuscript with help from S. M. All authors have given approval to the final version of the manuscript.

## Conflicts of interest

There are no conflicts to declare.

## Supplementary Material

SC-014-D3SC00686G-s001

SC-014-D3SC00686G-s002
