# Supplementary material for: Delayed luminescence guided enhanced circularly polarized emission in atomically precise copper nanoclusters
Source: Chem Sci. 2023 Apr 25;14(21):5593–601. doi: 10.1039/d3sc00686g (PMC10231326; doi:10.1039/d3sc00686g)
Supplement: SC-014-D3SC00686G-s001 [file SC-014-D3SC00686G-s001.pdf]

Electronic Supplementary Material (ESI) for Chemical Science.
This journal is © The Royal Society of Chemistry 2023

Supporting Information

Delayed Luminescence Guided Enhanced Circularly Polarized Emission in Atomically Precise Copper Nanoclusters

Camelia Dutta, Sonia Maniappan and Jatish Kumar*

Department of Chemistry, Indian Institute of Science Education and Research (IISER) Tirupati, Tirupati 517507, India

E-mail: jatish@iisertirupati.ac.in

Table of Contents

Sl. No.	Content	Page No.
1	Experimental Procedures	S2
2	Table S1. Table containing crystallographic parameters	S3
3	Fig. S1. Zeta potential plots of nanocluster solutions	S3
4	Fig. S2. PL decay of clusters in solution and solid states	S4
5	Fig. S3. g_{CD} spectra of nanoclusters in solution	S4
6	Fig. S4. Simulated structure and energy levels of nanocluster	S5
7	Fig. S5. Simulated absorption and CD plots of the nanoclusters	S5
8	Fig. S6. g_{lum} spectra of nanoclusters in solution and films	S6
9	Fig. S7. Evolution of PL and CPL with time	S6
10	Fig. S8. pH dependent UV-vis, PL, CD and CPL spectra	S7
11	Fig. S9. Temperature dependent PL and CPL spectra	S7
12	Fig. S10. Lifetime decay plot of clusters incorporated in PVA	S8
13	Fig. S11. CPL spectra of film collected from different positions	S8

Experimental Procedures

Materials

Poly vinyl alcohol, D and L-cysteine hydrochloride monohydrate were purchased from TCI chemicals. Copper(I) Iodide was purchased from spectrochem. Milli-Q purified water was used throughout the whole experiment. All the chemicals were used without further purification.

Characterization

The clusters were purified by using a centrifuge Z 366 K. Information about the surface charge of the as-prepared clusters were measured using Letesizer 500. FT-IR spectra was collected by PerkinElmer FT-IR Spectrometer. Information regarding the redox state of the metal in the clusters was collected by XPS measurements carried out using an ESCA Plus spectrometer (Omicron Nanotechnology Ltd., Germany) using a Mg Kα source. The powder XRD pattern was analysed using Rigaku Smart Lab 9 kW. Single crystal was analysed to obtain the crystal structure of the clusters using Bruker D8 VENTURE Super DUO Diffractometer with Photon-III Detector. The absorption and PL spectra of the clusters in solution and solid state were measured using Cary UV-vis Multicell Peltier and JASCO Spectrofluorometer- FP-8500, respectively. CD analysis was done on JASCO J-1500 CD spectrometer, and CPL measurements of the solution samples and films were done on JASCO CPL-300 spectrometer. Lifetime and absolute quantum yield were carried out on Edinburg FLS 1000 instrument.

Synthesis of (D/L-Cys)$_2$Cu$_4$I$_4$ nanoclusters

0.1 M CuI and 100 mM cysteine solutions were prepared separately using milli-Q water. 1 equivalent of the freshly prepared CuI solution was added into 3 equivalents of cysteine solution with vigorous stirring at the room temperature. The solution was stored overnight in dark to form bright yellow nanoclusters that precipitated in the vial. For removing the excess CuI and cysteine, the solution was centrifuged twice at 9500 rpm for 15 min, and the precipitate was collected. The as-prepared optically active Cu nanocluster solution was stored at 4 $^{\circ}$C. D/L-cysteine was used to obtain nanoclusters possessing opposite chirality. The nanocluster solution was diluted using milli-Q water for further studies.

Table S1. Crystallographic parameters of (D/L-Cys)$_2$Cu$_4$I$_4$ clusters.

	**(D-Cys)$_2$Cu$_4$I$_4$**	**(L-Cys)$_2$Cu$_4$I$_4$**
X-Ray Source	Monochromatic Mo	Monochromatic Mo
a	7.373(3)	7.155(7)
b	12.555(4)	7.155(7)
c	12.999(5)	12.562(18)
α	90^0	$92.42(3)^0$
β	$95.859(12)^0$	$92.42(3)^0$
γ	90^0	118.1035^0
Volume	1197.0(8)	565.4(11)
Z	1	2
R_1	6.85%	14.17%
R_{int}	8.05%	23.26%
Completeness	99.5%	92.4%
Method	Intrinsic Phasing 1	Patterson

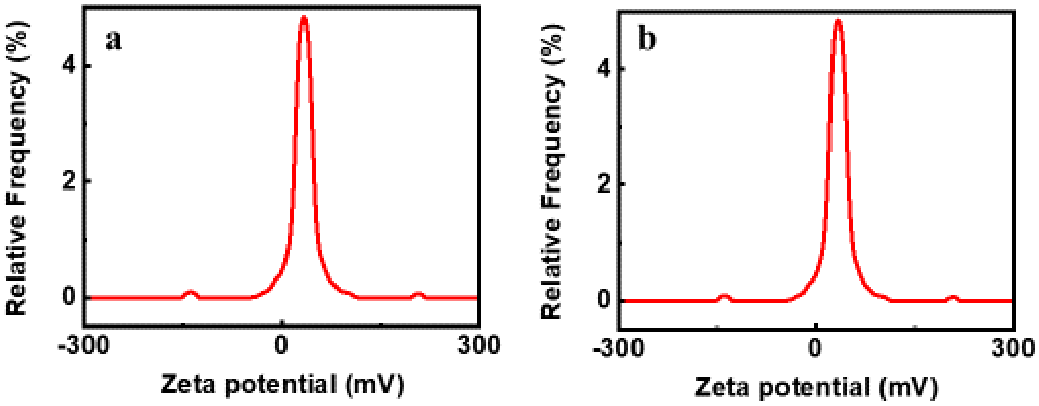

Figure S1. Zeta potential plots of (a) (L-Cys)$_2$Cu$_4$I$_4$ and (b) (D-Cys)$_2$Cu$_4$I$_4$ nanocluster solutions.

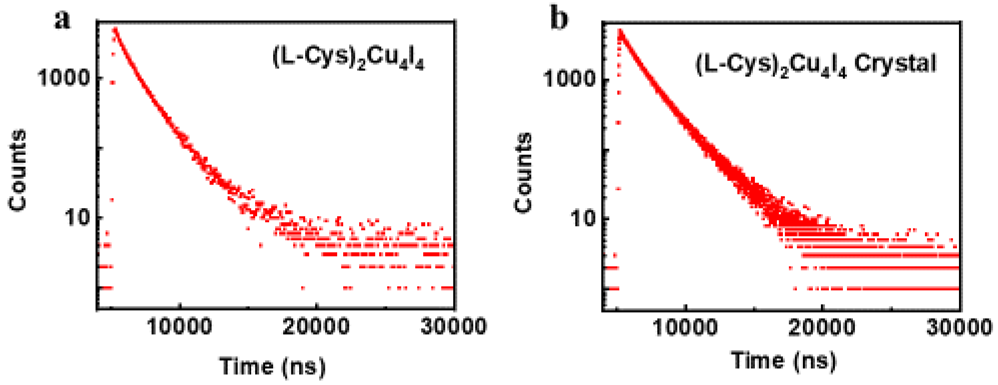

Figure S2. PL lifetime decay spectra of $(Cys)_2Cu_4I_4$ clusters in (a) solution and (b) crystals.

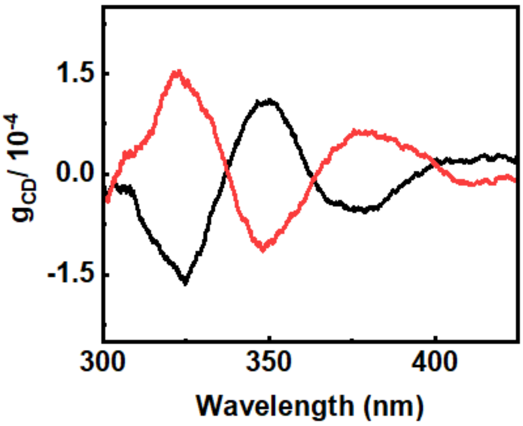

Figure S3. g_{abs} spectrum of (D-Cys)$_2$Cu$_4$I$_4$ (black trace) and (L-Cys)$_2$Cu$_4$I$_4$ (red trace) in solution.

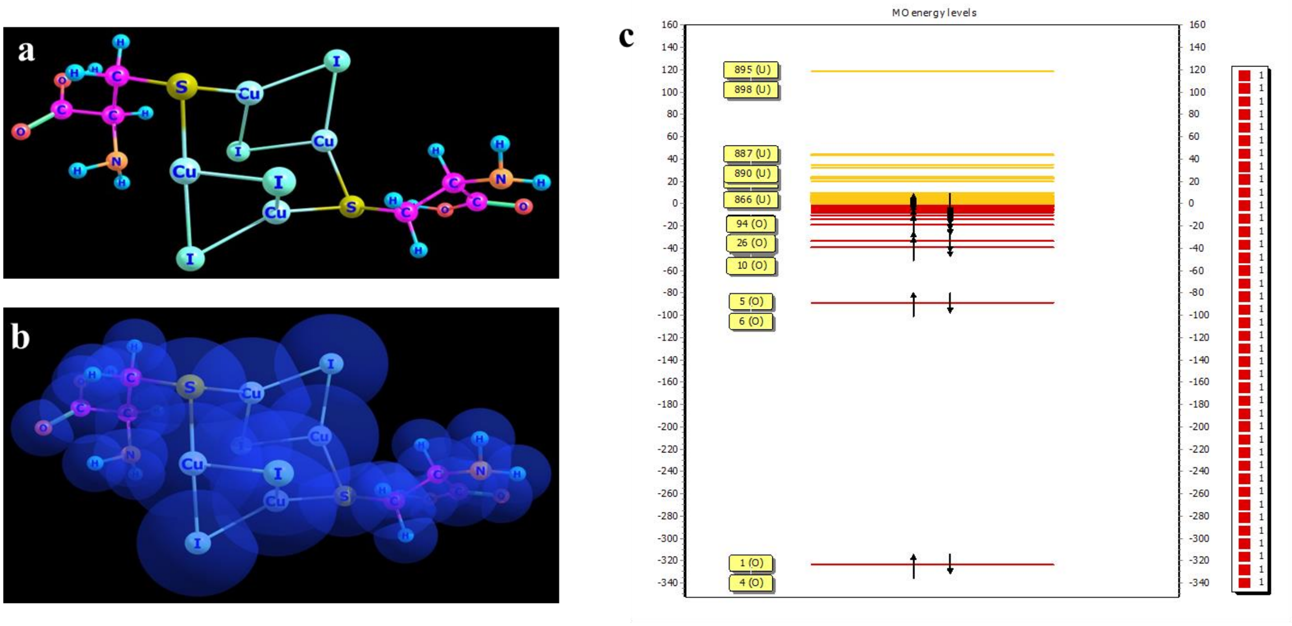

Figure S4. (a) Simulated structure and (b) the structure with van der Waals sphere of (D-Cys)$_2$Cu$_4$I$_4$ and (c) the corresponding molecular orbital (MO) diagram.

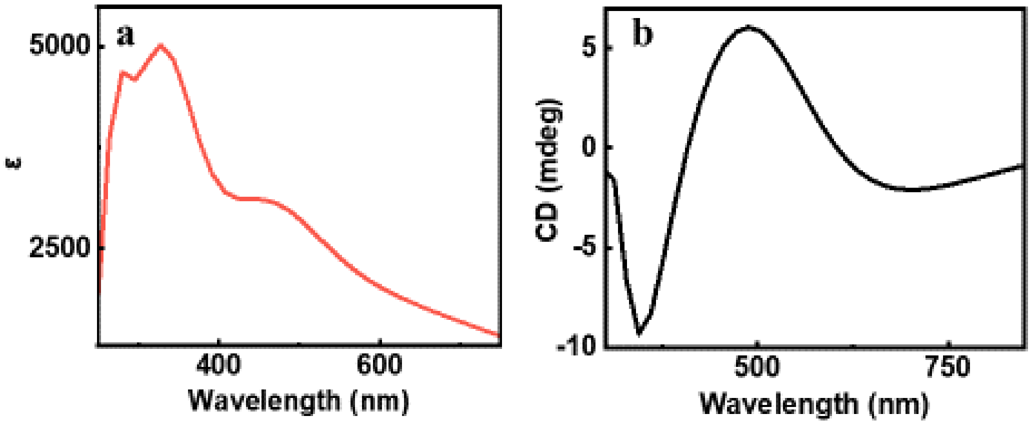

Figure S5. Simulated (a) absorption and (b) CD spectra of (D-Cys)$_2$Cu$_4$I$_4$ nanocluster.

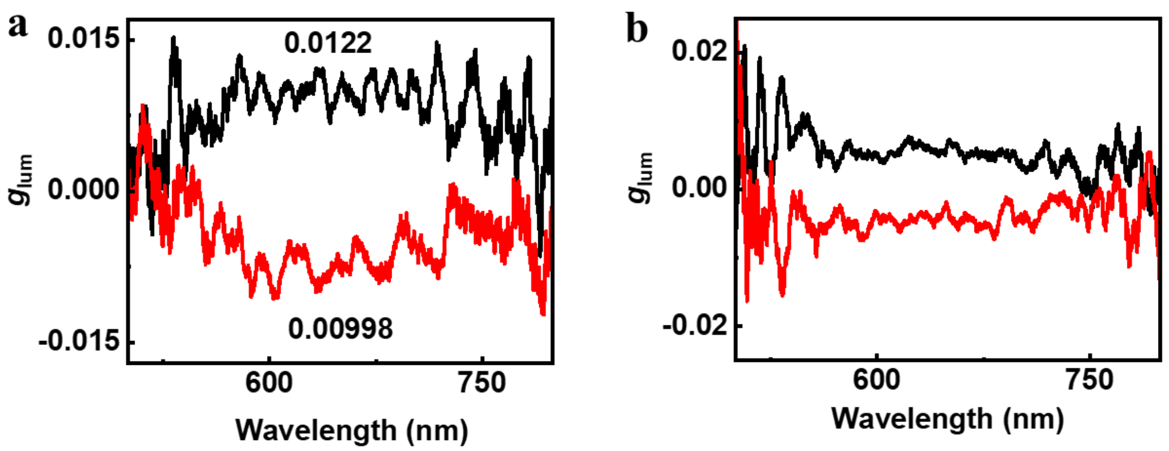

Figure S6. g_{lum} spectra of $(D\text{-}Cys)_2Cu_4I_4$ (black traces) and $(L\text{-}Cys)_2Cu_4I_4$ (red traces) nanoclusters in (a) solution state and (b) in PVA films.

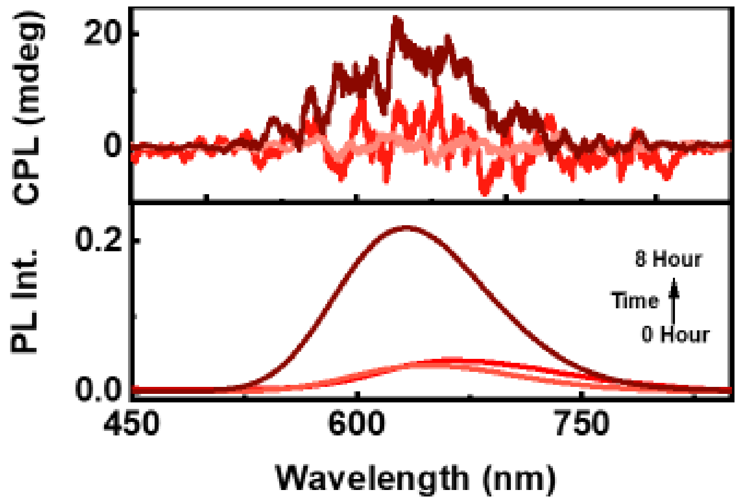

Figure S7. Evolution of CPL (top) and PL (bottom) spectra of $(D/L\text{-}Cys)_2Cu_4I_4$ clusters with time.

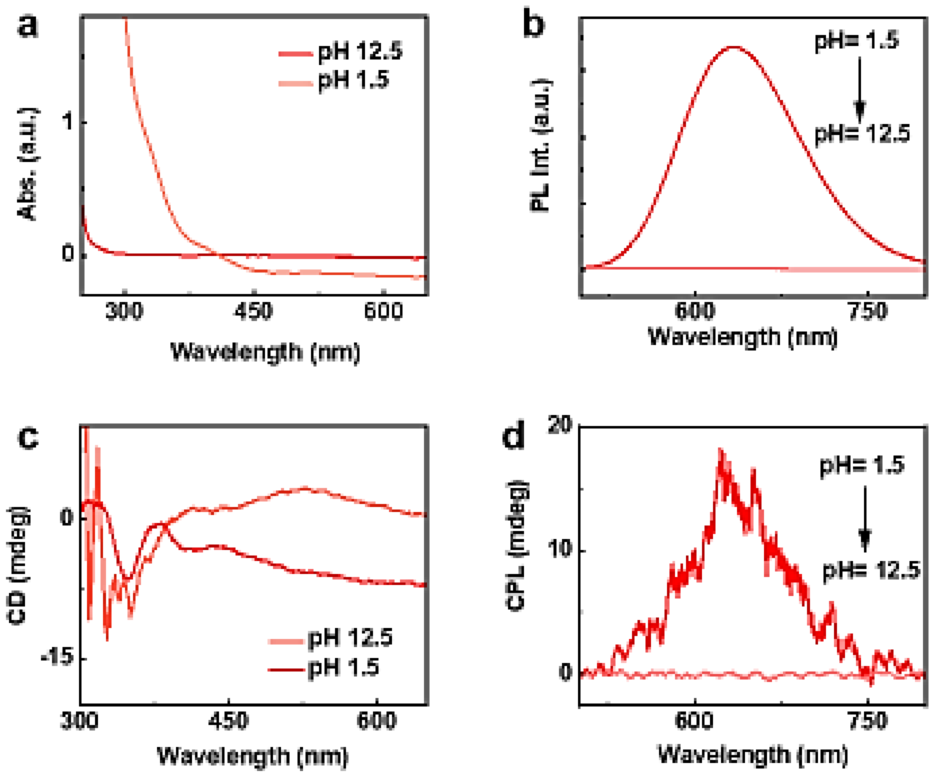

Figure S8. pH dependent (a) absorption, (b) CD, (c) PL and (d) CPL spectra of (D-Cys)$_2$Cu$_4$I$_4$ clusters.

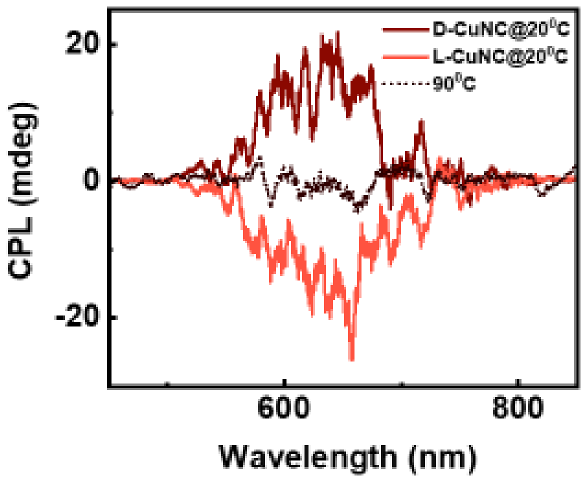

Figure S9. Temperature dependent CPL spectra of (D/L-Cys)$_2$Cu$_4$I$_4$ clusters.

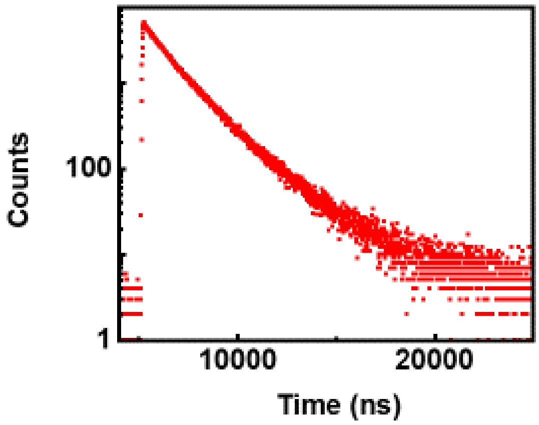

Figure S10. PL lifetime decay of $(D\text{-}Cys)_2Cu_4I_4$ clusters incorporated with PVA film.

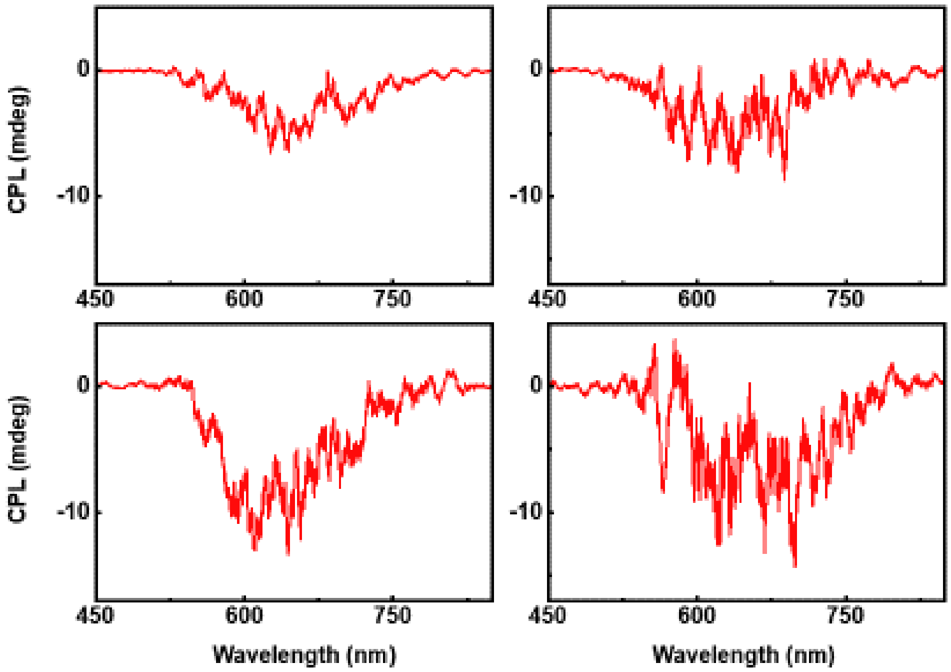

Figure S11. CPL spectra of the nanocluster incorporated PVA film collected after rotation of films at different angles and by flipping the films.
